# Improvement of Prediction Performance With Conjoint Molecular Fingerprint in Deep Learning

**DOI:** 10.3389/fphar.2020.606668

**Published:** 2020-12-18

**Authors:** Liangxu Xie, Lei Xu, Ren Kong, Shan Chang, Xiaojun Xu

**Affiliations:** ^1^ Institute of Bioinformatics and Medical Engineering, School of Electrical and Information Engineering, Jiangsu University of Technology, Changzhou, China; ^2^ Jiangsu Sino-Israel Industrial Technology Research Institute, Changzhou, China

**Keywords:** artificial intelligence, deep learning, fingerprints, quantitative structure-activity relationship, molecular descriptors

## Abstract

The accurate predicting of physical properties and bioactivity of drug molecules in deep learning depends on how molecules are represented. Many types of molecular descriptors have been developed for quantitative structure-activity/property relationships quantitative structure-activity relationships (QSPR). However, each molecular descriptor is optimized for a specific application with encoding preference. Considering that standalone featurization methods may only cover parts of information of the chemical molecules, we proposed to build the conjoint fingerprint by combining two supplementary fingerprints. The impact of conjoint fingerprint and each standalone fingerprint on predicting performance was systematically evaluated in predicting the logarithm of the partition coefficient (logP) and binding affinity of protein-ligand by using machine learning/deep learning (ML/DL) methods, including random forest (RF), support vector regression (SVR), extreme gradient boosting (XGBoost), long short-term memory network (LSTM), and deep neural network (DNN). The results demonstrated that the conjoint fingerprint yielded improved predictive performance, even outperforming the consensus model using two standalone fingerprints among four out of five examined methods. Given that the conjoint fingerprint scheme shows easy extensibility and high applicability, we expect that the proposed conjoint scheme would create new opportunities for continuously improving predictive performance of deep learning by harnessing the complementarity of various types of fingerprints.

## Introduction

Predicting molecular properties plays important roles in guiding drug discovery. In the last decade, applying machine learning to predict physical or chemical properties of molecular drugs gains great interest, especially since the emergence of deep learning (LeCun et al., 2015; Min et al., 2016; Shen et al., 2020). By converting molecules into computer readable formats, such as molecular descriptors, machine learning will map features through hierarchical non-linear functions to required outputs. Deep learning with matched input molecular descriptors has achieved breakthrough improvements in biology and chemistry fields, such as predicting quantitative structure-activity relationships (QSAR) (Butler et al., 2018), modeling absorption, distribution, metabolism, excretion and toxicity (ADMET) (Lei et al., 2017; Wu et al., 2019), virtual screening (Cereto-Massagué et al., 2015; Wang et al., 2020), drug design (Morrone et al., 2020), materials design (Xie and Grossman, 2018), chemical reactions (Grambow et al., 2020), and protein structure prediction (Senior et al., 2020), etc.

The accumulated experience in bioinformatics studies shows that the accurate predictions of machine learning heavily depend on the effective molecular representations (Schneider, 2010). Researchers from chemistry and biology field adopt many ways to design proper molecular descriptors, which requires strong experience and professional knowledge (Mater and Coote, 2019). Many types of molecular descriptors have been designed based on professional knowledge and specific demands. In the early days, the primary aim is to store and retrieve molecules, so that the molecular representations are compact and simple. The famous example is simplified input line entry system (SMILES) (Weininger, 1988; Weininger et al., 1989; Weininger, 1990). Later, the aim to search substructures drives to develop key-based fingerprints, such as molecular access system (MACCS) keys (Durant et al., 2002a). To meet growing need to model structure-activity and bioactivity, more effective fingerprints are designed, such as pharmacophore fingerprint and topological fingerprints. Recently, researchers are trying to incorporating 3D information in fingerprints for accurately predicting bioactivity of molecular drugs. More expert-designed fingerprints are continuously to be developed, such as 4D-fingerprints (Senese et al., 2004), molecular graphs (Kearnes et al., 2016), coulomb matrices and atomic coordinates (Sanchez-Lengeling and Aspuru-Guzik, 2018) or properties extracted from molecular dynamics simulations (Riniker, 2017).

**FIGURE 1:**
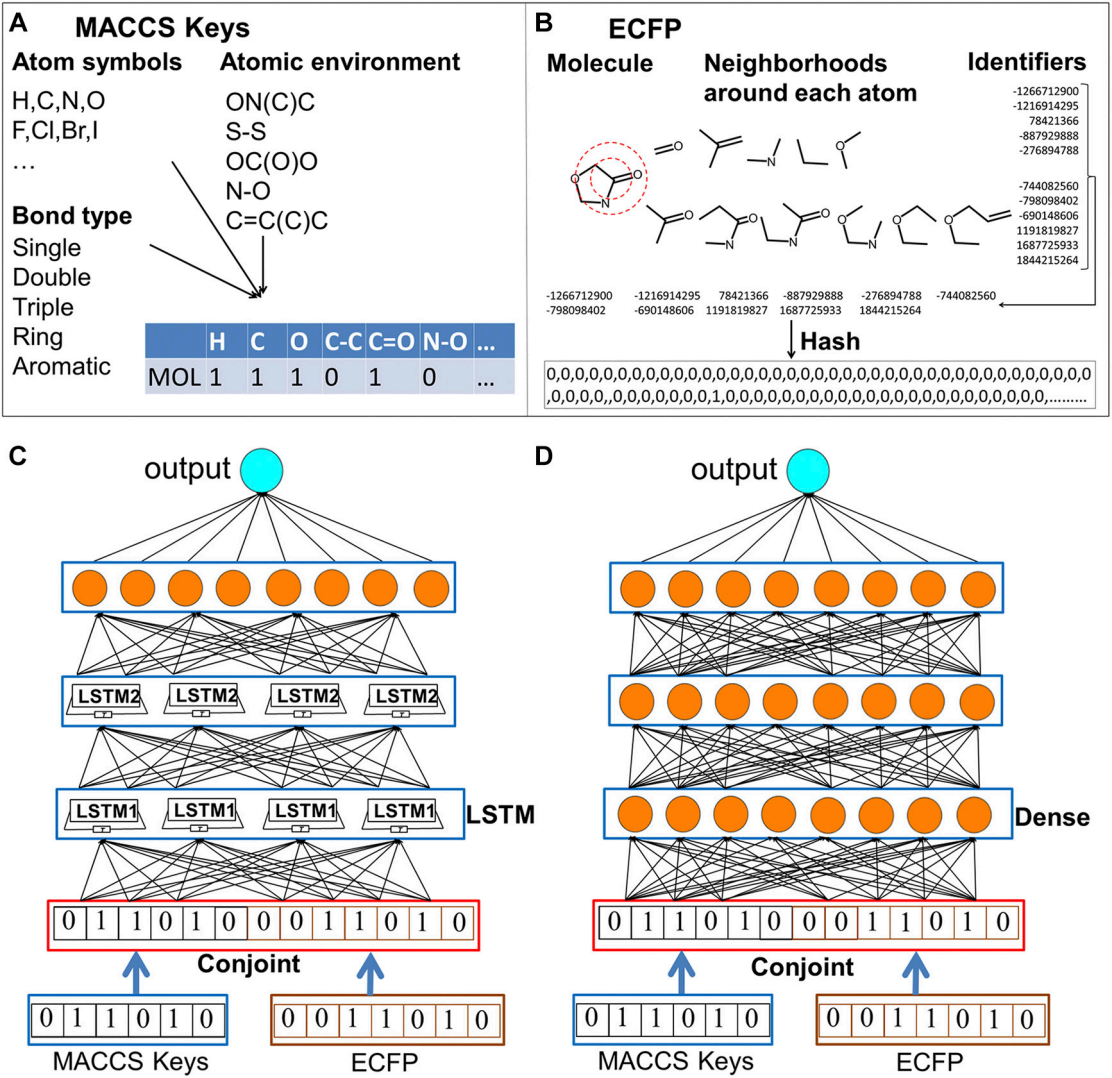
The schematic computing procedures of MACCS keys, ECFP fingerprints and the conjoint fingerprints for LSTM and DNN. One molecule is transformed to its feature (vector) space representation using MACCS keys and ECFP fingerprints. Two types of fingerprint are combined as conjoint fingerprint. Output of deep neural network is the predicted properties. **(A)** schematic computing procedures of MACCS keys; **(B)** schematic computing procedures of ECFP fingerprints; **(C)** LSTM trained with conjoint fingerprint; **(D)** DNN trained with conjoint fingerprint.

Though many types of molecular descriptors have been proposed, there is not “one size fits all” molecular representation. The domain expert-engineered molecular features sometimes becomes one main obstacle sitting on the road to deep learning (Chuang et al., 2020). Molecular descriptors represent molecular structures from holistic representations, such as molecular size, weight, molecular shape. In the contrast, molecular fingerprints describe the local aspect of chemical structures and exploding whether the presence of substructure patterns. Molecular fingerprints have been optimized based on the particular tasks. Existing molecular fingerprints encodes different information with preference to reproduce the best results for the designated tasks. The available fingerprints can be classified into five types: topological, geometrical, thermodynamic, electronic and constitutional fingerprints (Danishuddin and Khan, 2016). Several studies have been reported to check the performance of different fingerprint schemes (Duan et al., 2010; Riniker and Landrum, 2013; Wu et al., 2018). Each type of molecular descriptors combined with machine learning methods fits into the matching scope of applicability. Two most used molecular descriptors are MACCS keys and extended connectivity fingerprints (ECFP) (Rogers and Hahn, 2010a). MACCS key is the substructure key-based fingerprints, which includes predefined atom symbols, bond types, atom environment properties, atom properties (Figure 1) (Durant et al., 2002b; Cereto-Massagué et al., 2015). ECFP encodes local neighborhoods around each atom and bonding connectivity in molecules (Rogers and Hahn, 2010b). Both MACCS keys and ECFP have gained wide applications in similarity searching (Vilar et al., 2014; Cereto-Massagué et al., 2015), modeling QSAR (Glen et al., 2006; Myint et al., 2012), and predicting chemical reactivity (Sandfort et al., 2020). Wei group has adopted MACCS keys to encode protein and ligand pharmacological space and realized high predictive accuracy and improved high-throughput performance in drug discovery (Li et al., 2019). Recently, deep learning combined with ECFP fingerprint has been shown as a robust method for high throughput logP predictions, which obtained the root mean square error of 0.61 logP units and ranks as top quarter out of the 92 submissions in the sixth round of Statistical Assessment of the modeling of Proteins and ligands (SAMPL6) competition (Prasad and Brooks, 2020).

The importance of effective representations of molecules has been recognized when seeking higher accuracy of predicting results (Feinberg et al., 2020; Lui et al., 2020). Professional knowledge-based molecular descriptors will be one straightforward way but will bring great challenges for general users who are not familiar with computer techniques. Considering that the standalone molecular descriptors inherently cover parts of information of chemical molecules, we can develop new computing schemes to utilize the existing molecular descriptors. With realization of preference encoding in each molecular descriptor, combining two fingerprints together shows great potential in improving prediction performance in addition to design novel types of fingerprints (Tseng et al., 2012).

Though the combining of different classes of the molecular fingerprint was proposed by Tseng et al., only important molecular features were selected from the trial descriptor pool that is constructed from molecular representations (Tseng et al., 2012). The hybrid fingerprints with feature engineering have been actively employed in the field (Nisius and Bajorath, 2009; Wang et al., 2016). Features selection can be completed by genetic algorithms, least absolute shrinkage and selection operator (LASSO), or partial least square (PLS), etc. For example, Tseng applied genetic function approximation and multi-dimensional linear regression to select important descriptors from the entire descriptor pool (Senese et al., 2004). They also employed PLS to highlight import features from multiple descriptor pool in the predictive toxicology modeling (Su et al., 2012). Pérez-Castillo reported an automatic genetic algorithm to select features for binary classification when they built QSAR modeling (Pérez-Castillo et al., 2012). Algamal employed the adaptive LASSO method to study high-dimensional QSAR prediction of the anticancer potency. Feature selection methods are active not in QSAR modeling but also in machine learning fields (Algamal et al., 2015). Bajorath and coworkers extracted main features from MACCS keys, typed-graph distances (TGD) (Sheridan et al., 1996), typed-graph triangles (TGT) (Tovar et al., 2007) to form hybrid fingerprints for similarity searching (Nisius and Bajorath, 2009). In the latest research, Hou et al. also found that proper molecular descriptors selection was able to yield satisfied performance of machine learning (Fu et al., 2020; Jiang et al., 2020). These works proved that building proper hybrid fingerprints was one of important techniques for traditional machine learning methods (Cai et al., 2018). However, feature engineering is required to identify significant molecular features among molecular descriptor pools. Feature engineering process is a tedious and error-prone process and also requires professional knowledge (Wang and Bajorath, 2008; Hu et al., 2009).

Nowadays, deep learning shows capability of feature engineering and can automatically train algorithms to learn which fingerprints are important, leading to unique advantages in dealing with complex patterns of big data (Taherkhani et al., 2018). As been reported, automatically feature extraction endows deep learning with incomparable advantages in predicting physical and chemical properties of molecules in bioinformatics, chemistry, material science and drug discovery fields (Goh et al., 2017; Sanchez-Lengeling and Aspuru-Guzik, 2018; Yang et al., 2019; Jiang et al., 2020). Can we avoid feature engineering in the days of deep learning? Hop and coworkers proved that machine learned features outperformed than the domain expert engineered features (Hop et al., 2018). Tseng and coworkers reported that using raw data as molecular representations for deep learning can efficiently learn the most informative features (Chen and Tseng, 2020). As novel architectures have been developed, molecular descriptors even can be learned from low-level to high-level encodings of molecules during the training process (Kearnes et al., 2016; Winter et al., 2019). From the previsou success, we can find that more features lead to better prediction results by using deep learning.

Considering the limitations of standalone featurization and the automatic feature engineering ability of deep learning, we hypothesized that combining two complementary fingerprints rather than relying on expert engineering fingerprints may have room to improve performance of deep learning. As prospected by Tseng et al., the future of QSAR lies in developing new strategies that combine and use 1D through *n*D molecular fingerprints. Endeavors are worthy to be paid for optimizing the use of available descriptors or fingerprints (Tseng et al., 2012). By harnessing the automatic learning ability of deep learning, combining two types of fingerprints as input data for deep learning rather than manually feature engineering shows great potential in improving predicting performance. Combining multi-dimensional fingerprints preserves as much information as possible. The selecting fingerprints will be automatically completed during the training of machine learning or deep learning (ML/DL) methods by leveraging the learning ability of ML/DL methods. Under this context, we tried to circumvent feature engineering by selecting a proper pair of conjoint fingerprints and ML/DL methods. To facilitate practical applications of deep learning, we adopt two well established molecular fingerprints, MACCS keys and ECFP fingerprints, to construct conjoint fingerprints for deep learning. MACCS keys contain the constituent elements and predefined substructural keys of molecules while lacking substructure connectivity. ECFP contains bonding connectivity and topological features. These two types of molecular fingerprints can provide supplementary information in predicting physicochemical properties. However, the evaluation of combining of MACCS keys and ECFP fingerprints has not been reported.

In this study, we validate the performance of conjoint fingerprints by using three classic machine learning methods (RF, SVR, and XGBoost) and two deep learning methods (LSTM and DNN) in the predicting the logarithm of partition coefficient of a molecule between water and the lipid phase (abbreviated as logP), and the binding affinities of protein-ligand. To demonstrate the generalizability of the proposed conjoint fingerprint, we conducted the regression task on three tasks: predicting logP of DrugBank database (Wishart et al., 2018), predicting logP of the Lipophilicity dataset that is collected in the MoleculeNet (Wu et al., 2018), and predicting binding affinities for protein-ligand complex in the PDBbind database (Liu et al., 2014). It is expected that deep learning can automatically learn the proper representations from conjoint fingerprints, which will overcome the limitations of feature engineering in machine learning methods.

## Materials and Methods

### Data Preparation

DrugBank database. The molecular structures and the corresponding logP values were obtained from DrugBank database (Wishart et al., 2018). DrugBank database collects the detailed drug data and the comprehensive drug target information. The logP value is one of most concerned properties of drug molecules, which measures the solubility, absorption, and membrane penetration of drug molecules in the tissues. The DrugBank database contains two subsets: the FDA approved drug molecules, which will be named as “Approved” subset, and all molecules including potential drugs under study, which will be referred as “All” subset in this study. Until 2020, there are 13,566 drug entries. Among them, 2011 and 8,656 drug molecules contain logP value entries in the “Approved” and “All” subset. In current study, 20% of data were randomly selected as test set and the remaining data were further separated as training and validation dataset with the ratio 4:1 in the hyperparameter optimization using Grid Search with cross-validation (GridSearchCV) method (GridSearchCV, 2020). The dataset was split using the same random seed to keep reproducibility for different validated models.

Lipophilicity dataset. We also selected the Lipophilicity dataset that is collected in the MoleculeNet to present the general applicability of conjoint fingerprints in a different dataset. The Lipophilicity dataset consists of the experimental value of the octanol/water distribution coefficient, which is curated from ChEMBL database. Based on this high-quality dataset, we further validated the performance of standalone and conjoint fingerprints when using 5 ML/DL methods. For comparison, we also computed SlogP by using one traditional logP prediction approach proposed by Wildman-Crippen logP prediction approach (Wildman and Crippen, 1999), which is implemented in RDKit (RDKit, 2017). Moreover, we checked the “random” splitting and “scaffold” splitting effect on the performance. “Scaffold” splitter in the DeepChem was used to split the Lipophilicity dataset into training and test subsets (DeepChem, 2018).

PDBbind dataset. The refined subset of PDBbind was selected because the refined subset contains high quality experimental dissociation constant or inhibition constant (referred as pKi) data for the reasonable number of protein-ligand structures. We used the MACCS keys and ECFP fingerprint to predict pKi of the refined subset of PDBbind. The bound ligands and the binding pockets of protein within 4.5 Å of ligand were converted into MACCS keys and ECFP fingerprint, respectively. Water molecule and metal ions within the pocket were deleted due to the technical limitations of RDKit. 4,752 structures were successfully converted to fingerprints. The conjoint fingerprints were built by concatenating MACCS keys and ECFP fingerprint strings. The major focus is on the performance comparison between separated and conjoint fingerprint, therefore the same set of hyperparameters that optimized for DrugBank was adopted.

Fingerprint conversion. The molecular structures and logP were extracted from the SDF files of DrugBank database. The molecules were converted from Cartesian coordinates into vector space representation. Specifically, MACCS keys use a dictionary to check whether the atom types and substructure exist. MACCS keys only cover information of atom and bond types for one molecule and provide limited connecting information in chemical molecules. While ECFP includes the information of how atoms bonded with each other but does not include the chemical properties of each atoms. The combining of MACCS keys and ECFP fingerprints can provide supplementary information in the predicting physicochemical properties. For MACCS keys, the type with 166 keys is the most commonly used in virtual screening. Therefore, each drug molecule was converted into a 166-bit structural MACCS key by checking whether the substructures exist. MACCS keys were computed by using RDKit (RDKit, 2017). ECFP fingerprints analyze the bonded structural information within the circular radius of atoms. The local structural information around each atom was converted into integer identifiers and then hashed to a bit vector. A radius of two bond lengths was usually used in ECFP. A fixed number of vectors of 2048-bit circular fingerprint were adopted in this study. The ECFP fingerprints were converted by DeepChem open-source package which was developed in Pande group (Ramsundar et al., 2017).

Machine learning and deep learning algorithms were trained with conjoint fingerprints along with MACCS keys and ECFP fingerprint separately. The conjoint scheme was built by concatenating two strings into one input string (Figure 1). The conjoint fingerprint is expected to be more informative by covering both substructural and topological information. The impact of the conjoint fingerprint on performance will be checked in comparison with MACCS keys and ECFP using five learning algorithms.

Random forests. Random forests (RF) was normally selected as a baseline to compare with deep learning methods. RF attracts much interest in QSAR/QSPR studies because it is not sensitive to the hyperparamters. RF outstands from other machine learning methods with advantages of high accuracy (Breiman, 2001). RF is an ensemble prediction method, which consists of many individual decision trees and the final results are averaged over each individual tree. RF can complete random feature selections in the trees. The difference between RF and DNN is that RF split the whole feature into fragment for each individual tree while DNN can simultaneously process whole features. The number of estimators, tree depth and the number of leafs were selected based on GridSearchCV method.

Support vector regression. The support vector machine (SVM) is designed to classification problems. To do regression, SVR tries to find a hyperplane with the minimized sum of distance from data to the hyperplane. The hyperplane is the combination of functions that parameterized by support vectors. SVR is one popular machine learning methods in QSAR/QSPR with advantages in modeling nonlinear problems. In the “RBF” kernel, “C” is the regularization parameter, which is inversely proportional to the strength of the regularization. A higher “C” value leads to lower tolerance toward to misclassification of training data. “Gamma” is the coefficient of “RBF” kernel, which is inversely proportional to the variance of Gaussian distribution. It controls how far the influence of a selected support vector reaches. The value of “C” and “gamma” was chosen from a GridSearchCV method using ECFP fingerprint. The “RBF” kernel function with “C” equals to five and “gamma” equals to 0.015 was adopted in this study.

Extreme gradient boosting. Extreme gradient boosting (XGBoost) model is recognized as a new generation of ensemble learning model. It is developed under the Gradient Boosting framework and is developed sequentially in a stagewise additive model. It can solve many data science problems with improved speed and accuracy. It has dominated in machine learning and Kaggle competition with higher performance and robust speed. XGBoost has been reported to achieve comparative performance than deep neural network (Sheridan et al., 2016).

Architecture of long short-term memory network. Long short-term memory network (LSTM) is improved based on the recurrent neural network (RNN). The advantage of LSTM is its ability to process sequence information with long-term dependency information. LSTM may be benefited from conjoint fingerprints, where two types of fingerprints are kept. The general architecture of LSTM unit is composed of an input gate, a forget gate, an output gate and a memory block. The forget gate is used to decide what information will be forgot from previous cell states. The input gate controls how much information will be kept for new cell states. The output gate determines the output information for new state. LSTM passes information selectively through gating mechanism by incorporating the memory cell that learns when to forget previous hidden states and when to update new hidden states. In this study, we adopted two LSTM layers, which were connected sequentially with one dense layer and one output layer. The time step was set to 1. The output dimension of the first LSTM layer was set to the same dimension of input data. To the best of our knowledge, it is the first time to implement LSTM in predicting logP value for drug molecules.

Architecture of deep neural network. Deep neural network (DNN) is a prototypical deep learning architecture. The important advantage of using DNN is that it can extract useful features from the raw input data. The typical DNN contains three parts: input, hidden and output layer. Each layer contains a set of neurons. We trained different DNN which varied in the size and number of neurons of hidden layers in their architecture. The number of neurons, batch size, epoch number, dropout rate and activation were searched over the hyperparameter space using K-fold cross validation over the training set using GridSearchCV method. In the study, five-fold was employed for the training dataset during hyperparameter optimization. Specifically, the number of neurons of hidden layer used is 10, 20, 40, 50, 60, 100, 300, and 500. Activation function of “softsign”, “rectified linear unit (relu)”, “linear” and “tanh” were tested each by each. The optimizer of “adaptive moment estimation (Adam)” with the default learning rate of 0.001 was employed in this study because Adam optimizer uses the adaptive learning momentum and also performs efficiently (Kingma and Ba, 2015). To reduce the overfitting, the dropout rate from 0 to 0.6 with interval of 0.1 was optimized using GridSearchCV method. During training and validation, the batch size and number of epochs were searched. The processes were repeated 20 times to calculate ensemble averages.

Consensus model. We also build the hybrid network models using the consensus model idea of DeepDTA (Öztürk et al., 2018). The consensus model has been reported to provide superior performance than single model in some recent researches (Öztürk et al., 2018; Fu et al., 2020). Different from Hou’s work (Fu et al., 2020), we trained machine learning methods with two standalone fingerprints rather than using different types of methods. Consensus model was constructed from different inputs for the same machine learning methods and can reduce statistical bias brought from single learning algorithm.

Performance evaluation. The mean squared errors (MSE) were calculated based on the following equation as the loss function during hyperparameter tuning.

$$MSE=\frac{\sum^{\text{​}}{\left(\log P^{exp}-\log P^{cal}\right)}^{2}}{n}$$

The root mean squared errors (RMSE) were computed to present the accuracy of examined learning algorithms. The predicting performance was checked based on a linear correlation between predicted and true logP value in DrugBank database on the given set of drug molecules. The overall agreement between experimental and predicated value was assessed by computing Pearson correlation coefficient according to the following equation

$$R^{2}=\frac{\sum^{\text{​}}\left(\log P^{exp}-\bar{\log P^{exp}}\right)\left(\log P^{cal}-\bar{\log P^{cal}}\right)}{\sqrt{\sum^{\text{​}}{\left(\log P^{exp}-\bar{\log P^{exp}}\right)}^{2}\sum^{\text{​}}{\left(\log P^{cal}-\bar{\log P^{cal}}\right)}^{2}}}$$

The Keras-2.2.2 was used to build the models and to optimize hyperparameters. Tensorflow version 1.14 and scikit-learn version 0.20 were used for training and evaluating for five learning algorithms.

## Results

### The Distribution of logP in DrugBank Database

**FIGURE 2:**
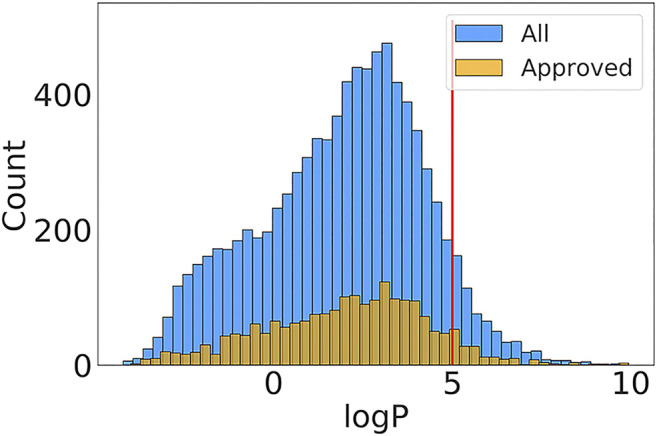
The logP value distributions in “Approved” and “All” subset of DrugBank. The red line represents the upper value of logP in Lipinski’s rule of five invented by Pfizer.

The logP is the partition coefficient of a chemical molecule between water and lipid phase, which measures the ability of molecular absorption and excretion. The computational methods of logP prediction can be classified into two major categories: substructure-based and property-based methods. Mannhold et al.’s review summarizes the available logP prediction approaches and provides benchmarked results for 30 methods (Mannhold et al., 2009b). The logP has been used to estimate transport ability of molecules through membranes and metabolisms in tissues, which has been included in the Lipinski’s rule of five. Considering the importance of predicting logP, we selected the chemical molecules and the corresponding logP values in DrugBank. The empirical logP values ranged from -4.21 to 9.72. the proportion of 93% of drug molecules shows logP smaller than 5 (Figure 2). The distribution is consistent with Lipinski’s rule of five, which states that the logP should ideally be not greater than five for orally bioavailable druglike small molecules.

**FIGURE 3:**
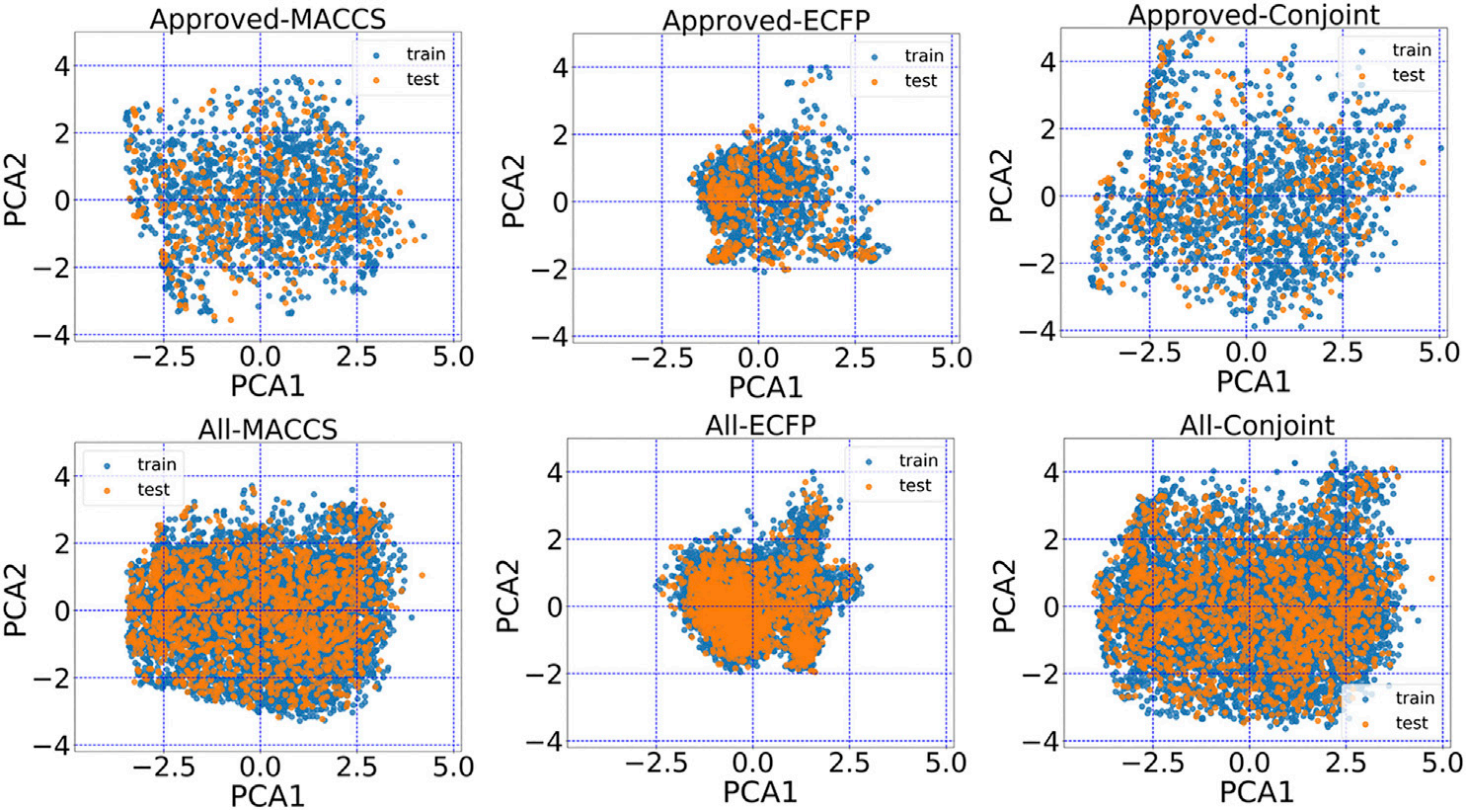
The principal component analysis plot of the first two principal components based on MACCS keys, ECFP and the conjoint fingerprint.

The encoded chemical space of MACCS keys, ECFP and conjoint fingerprints can be projected on the principle components to aid visualization. From principle components analysis (PCA), the conjoint fingerprint shows the more degree of dispersion in comparison with MACCS keys and ECFP. As shown in Figure 3, MACCS keys and ECFP distributed around a local region and the represented chemical space was not as wide as the conjoint fingerprint, implying more chemical space was kept in conjoint fingerprints. The training set and test set share the same distribution and may guarantee reasonable prediction performance.

### Hyperparameter Optimization

Tuning hyperparamters is critical for the predicting performance. We conducted Grid Search with cross-validation (GridSearchCV) method to tune hyperparamters with 5-fold cross validation scheme by using “Approved” and “All” data subset of DrugBank. Each data set was further separated as training, validation and test sets. We examined the predicting performance by using MACCS keys, ECFP and the conjoint fingerprints. The negative of mean squared error acted as mean score to evaluate the results as shown in Supplementary Figures S1–S4. Clearly, the optimal hyperparameters should be tuned in a statistical way as the mean score fluctuated for each examined parameter. The parameters were chosen based on the 20 round cross validations rather than a chance encounter. The selected parameters were summarized in Supplementary Tables S1 andS2.

### Conjoint Fingerprint Improved Predictive Accuracy

**FIGURE 4:**
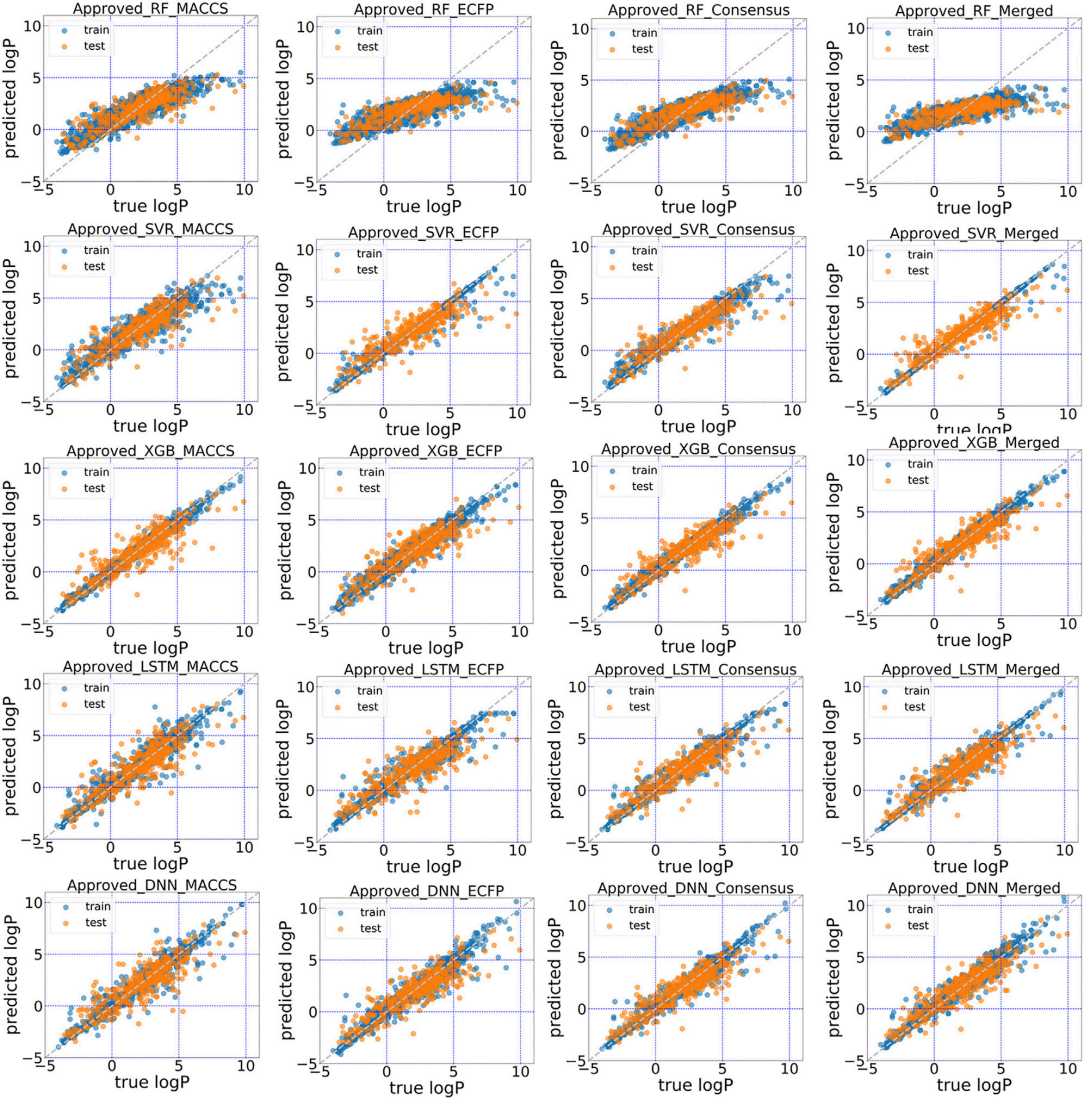
Scatter plot of the predicted logP vs. stored data in “Approved” dataset of DrugBank for five models with MACCS, ECFP, consensus model and the conjoint fingerints.

**FIGURE 5:**
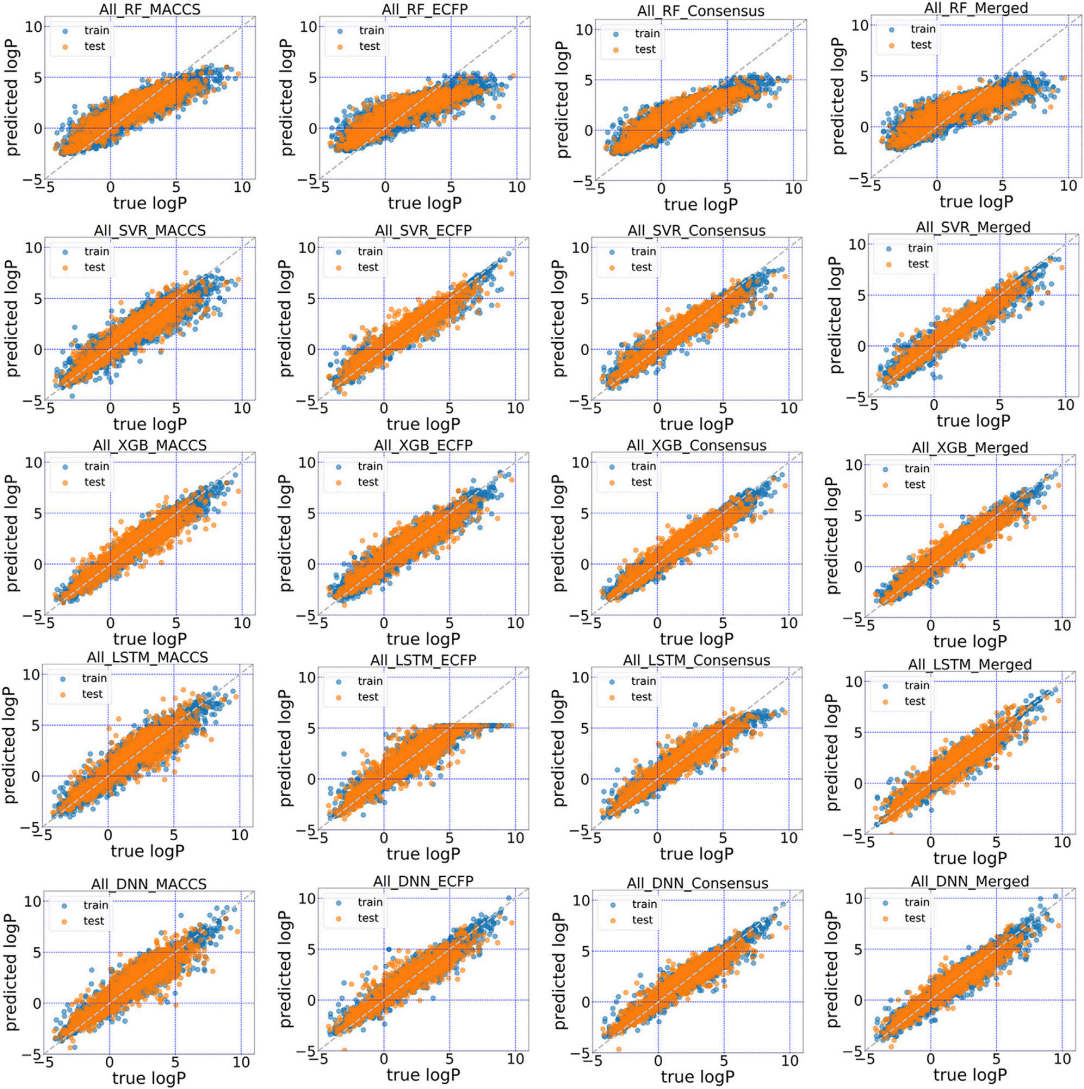
Scatter plot of the predicted logP vs. stored data in “All” dataset of DrugBank for five models with MACCS, ECFP, consensus model and the conjoint fingerprints.

The predictive performance for unrecognized molecules was validated in the test subsets using in total five machine learning and deep learning algorithms. The scatter plots of predicted logP against stored logP value in DrugBank were shown in Figures 4, 5. Clearly, conjoint fingerprint can provide the better distribution and higher predictive accuracy for the test set than that of MACCS keys and ECFP by using SVR, XGBoost, LSTM and DNN. RMSE was calculated to evaluate the overall error for the test set and was shown in Table 1. Overall, “All” subset displayed smaller root mean squared error (RMSE) than “Approved” subset. From our results, the obvious improvement is observed when dataset changes from “Approved” to “All” subset. If there are more high-quality data, the predictive performance of deep learning can be further improved.

**TABLE 1:** Root mean square error and distribution for each validated fingerprints.

		Approved					All				
				% Of molecules within error range					% Of molecules within error range		
Methods	Fingerprint	*R* ^2^	RMSE	<0.5	0.5–1	>1	*R* ^2^	RMSE	<0.5	0.5–1	>1
RF	MACCS	0.85	1.04	32.3	29.8	37.9	0.89	0.87	37.0	28.5	34.5
RF	ECFP	0.80	1.24	28.8	21.6	49.6	0.86	1.02	31.7	26.4	41.9
RF-Cons	MACCS + ECFP	0.83	1.01	30.8	25.6	43.67	0.87	0.91	35.4	27.7	36.9
RF	Conjoint	0.79	1.35	24.1	22.8	53.1	0.85	1.07	30.4	24.8	44.8
SVR	MACCS	0.89	0.82	43.9	28.3	27.8	0.92	0.63	52.3	27.0	20.6
SVR	ECFP	0.89	0.79	47.9	25.1	27.0	0.94	0.56	57.8	26.0	16.2
SVR-Cons	MACCS + ECFP	0.89	0.72	49.1	29.0	21.9	0.93	0.52	60.2	26.8	13.0
SVR	Conjoint	0.92	0.69	52.4	27.3	20.4	0.96	0.48	63.1	25.8	11.1
XGB	MACCS	0.90	0.75	47.9	26.8	25.3	0.93	0.61	53.7	27.1	19.2
XGB	ECFP	0.88	0.81	42.9	27.1	30.0	0.93	0.64	50.0	29.5	20.5
XGB-Cons	MACCS + ECFP	0.89	0.71	50.1	26.6	23.3	0.93	0.55	56.6	28.0	15.4
XGB	Conjoint	0.91	0.69	52.1	27.3	20.6	0.95	0.52	60.0	26.2	13.8
LSTM	MACCS	0.87	0.82	44.9	27.8	27.3	0.92	0.69	48.3	28.3	23.3
LSTM	ECFP	0.88	0.89	39.5	29.3	31.3	0.91	0.66	51.0	26.8	22.2
LSTM-Cons	MACCS + ECFP	0.87	0.74	47.9	26.3	25.8	0.92	0.57	56.4	27.2	16.4
LSTM	Conjoint	0.91	0.75	46.2	28.5	25.3	0.95	0.54	59.6	24.9	15.5
DNN	MACCS	0.88	0.81	45.7	25.3	29.0	0.91	0.70	47.2	30.0	22.3
DNN	ECFP	0.89	0.81	43.4	28.8	27.8	0.94	0.60	53.2	29.9	16.9
DNN-Cons	MACCS + ECFP	0.89	0.72	49.9	25.8	24.3	0.92	0.56	55.6	29.1	15.3
DNN	Conjoint	0.92	0.69	47.6	30.0	22.4	0.96	0.53	57.4	29.3	13.3

To facilitate the comparison between different schemes, the same set of hyperparameters that selected based on MACCS keys was used for conjoint fingerprint. From Table 1, we can notice that the smallest RMSE for “Approved” and “All” dataset were 0.686 and 0.475, which obtained from XGBoost and SVR with conjoint fingerprint, respectively. Conjoint fingerprint increased prediction accuracy for SVR, XGBoost, LSTM, and DNN when predicting logP values.

Furthermore, the predictive accuracy was quantified using the deviation counting statistics. We classified the prediction accuracy using the same criteria used by Tetko (Mannhold et al., 2009a), the deviation between predicted and true logP in the range of 0.0–0.5 as considered as “acceptable”, 0.5–1.0 as “disputable”, and larger than 1.0 as “unacceptable”. Therefore, counting statistics for RMSE was classified into three regions. For “All” dataset, the percentages within “acceptable” range took up to 63.1% when using conjoint fingerprints in SVR, which was higher than that of each standalone fingerprint (52.3 and 57.8% for MACCS and ECFP). Except RF, other methods also achieved similar conclusion. The results demonstrated that the conjoint fingerprint could improve predictive performance and also showed satisfactory generalization ability in predicting logP values of drug molecules. Overall, conjoint fingerprint reproduced the least RMSE than each standalone fingerprint even without optimal hyperparameters.

### Conjoint Fingerprint Boosted Overall Performance

**FIGURE 6:**
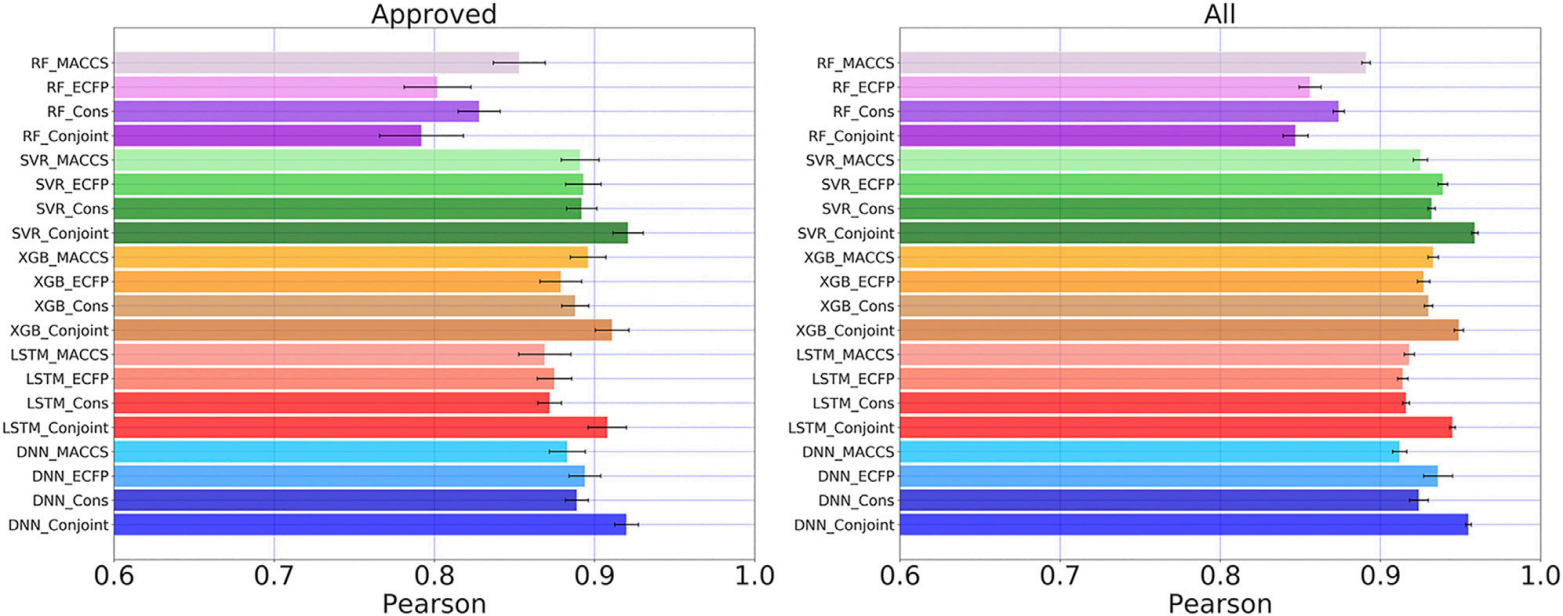
Pearson coefficients for “Approved” and “All” dataset calculated by five learning algorithms using MACCS keys, ECFP, consensus model and the conjoint fingerprints. The similar set of color represents one learning algorithm using different molecular fingerprints. Error bars were computed from 20 times individual training and testing processes.

We compared the overall predicting results among RF, SVR, XGBoost, LSTM, and DNN. The Pearson coefficients of the same test set were calculated for all examined methods. The generalization ability is another important indicator to examine the predictive performance of deep learning. We run 20 individual training by randomly separate dataset into training and testing set. The average Pearson coefficients and error bars were computed to present generalization ability. From Figure 6, the conjoint fingerprint improved predictive performance over MACCS keys or ECFP, suggesting that the conjoint fingerprints achieved complementarity of two types of fingerprints. DNN generally outperformed over other methods when predicting logP values in “Approved” subset. The Pearson coefficient of DNN with conjoint fingerprint reached to 0.910. When data becomes more, the kernel-based method, SVR, showed remarkable predictive performance by reproducing the highest Pearson coefficient of 0.959 in “All” subset. With enough data, SVR displays increasingly performance in treating nonlinear problems and presents better generalization performance. In general, the improvements benefited from the conjoint fingerprint have been realized in SVR, XGBoost, LSTM, and DNN. In this study, we adopted the same set of hyperparamters tuned based on MACCS keys. The performance can be improved with fine-tuned hyperparameters (see Supplementary Table S3 for more information). Conjoint fingerprints increase prediction accuracy, implying that the logP of molecules is relevant with both substructures and its neighboring atomic environment. Therefore, the standalone fingerprints cannot surpass the conjoint fingerprints.

### Comparison Between Ensemble Learning and Deep Learning

RF and XGBoost are the ensemble learning methods. The remarkable performance of XGBoost has been demonstrated in previous studies (Lei et al., 2017). We also obtained consistent results as shown in Figure 6. For RF, the Pearson coefficient even decreased for conjoint fingerprint. This is consistent with previous studies that the feature engineering is required for traditional machine learning method (Solorio-Fernández et al., 2020). As has been pointed by Hou et al., the machine learning methods displayed different prediction capabilities and some machine learning methods showed comparative performance as deep learning (Fu et al., 2020). Therefore, prediction models should be adopted on a case-by-case basis.

RF employs different approaches to process input fingerprints. RF consists of many decision trees and it splits the fingerprints for each individual tree. Each tree of RF samples parts of input fingerprints and cannot harness the complementarity information from conjoint fingerprint. The presence of irrelevant or redundant fingerprints even reduces the predictive accuracy for machine learning methods (Cai et al., 2018). While DNN or LSTM can process all input fingerprints at the same time, from which it automatically learns and identifies useful features. The results demonstrate that the proposed conjoint fingerprints can be combined with deep learning to improve predicting accuracy by taking full advantages of automatic feature engineering in DNN and LSTM.

### Comparison Between Conjoint Fingerprint and Consensus Model

**FIGURE 7:**
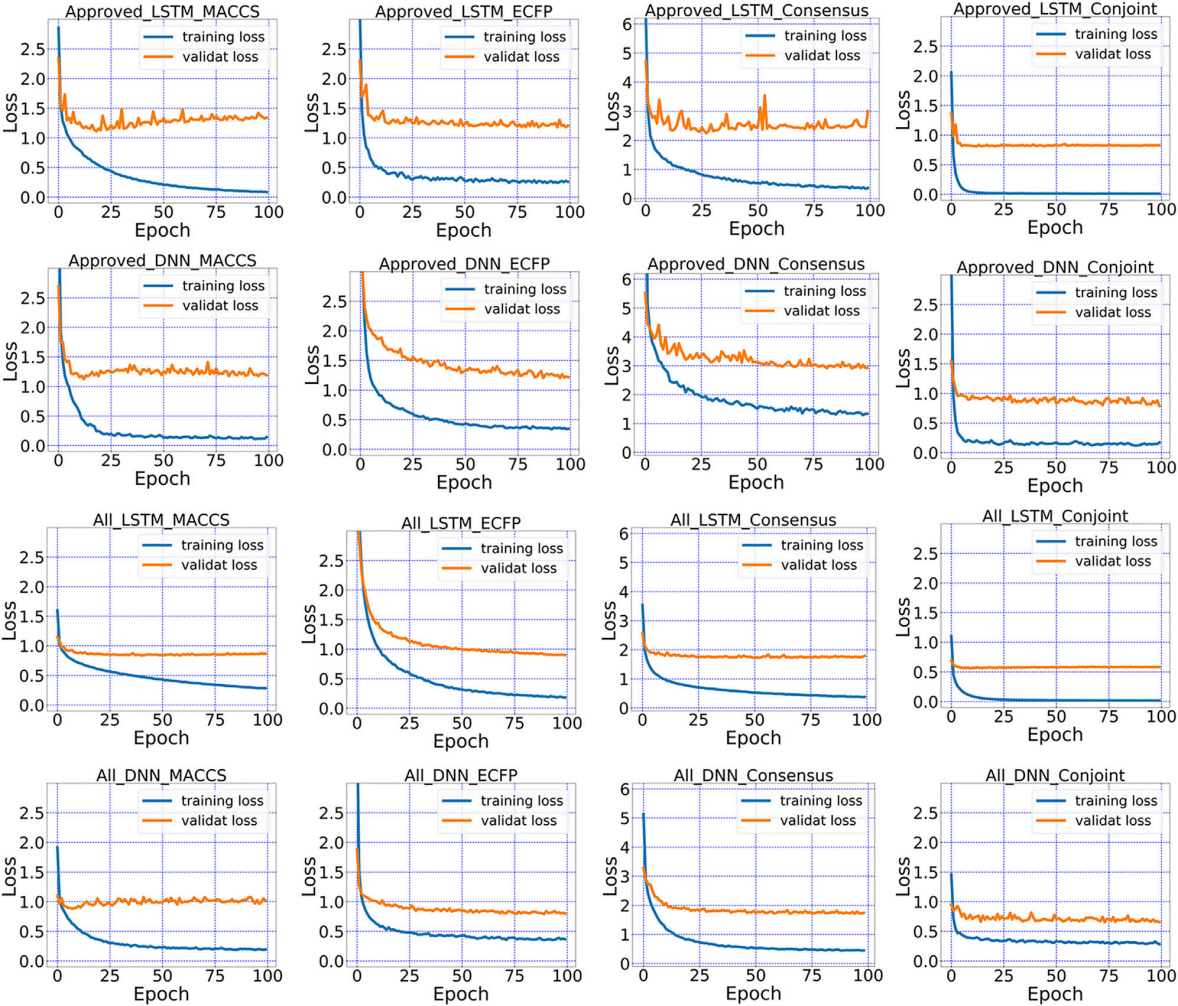
The loss in the training and validation dataset for MACCS keys, ECFP, the conjoint fingerprints.

Consensus model showed superior performance than each standalone method but did not surpass the performance of conjoint fingerprint. The loss can be tracked during the training process of LSTM and DNN. As revealed in Figure 7, MACCS keys and ECFP showed the larger deviation between training and validation subsets in consensus model than that of the conjoint fingerprint. Conjoint fingerprints reproduced the least deviation for both “Approved” and “All” subset. The deviation decreased from 0.827 to 0.583 for LSTM when dataset changed from “Approved” to “All” (see Supplementary Table S4 for more information). The loss value of consensus model in “All” subset was 1.772 while it was 0.656 for the conjoint fingerprint for DNN. From Figure 7, the loss for LSTM and DNN with conjoint fingerprint leveled off within 20 epochs. Consensus models required more training cycle. The loss did not level off until after ∼80 epochs for consensus models. As revealed from the training and validation loss, conjoint fingerprints required less training cycle and increased robustness than consensus model.

Conjoint fingerprints scheme outperformed over consensus model that uses standalone fingerprint. The reason may be that the input was trained separately in the consensus model and some information may be lost along with dimension reduction during the training through trees or neural network layers. The results are controlled by “buckets effect”, which limits further improved predictive accuracy. In contrast, the conjoint fingerprint conserves all information, which can be leveraged by deep learning to reproduce more accurate results.

### The Generalizability of Conjoint Fingerprints for Other Regression Tasks

**FIGURE 8:**
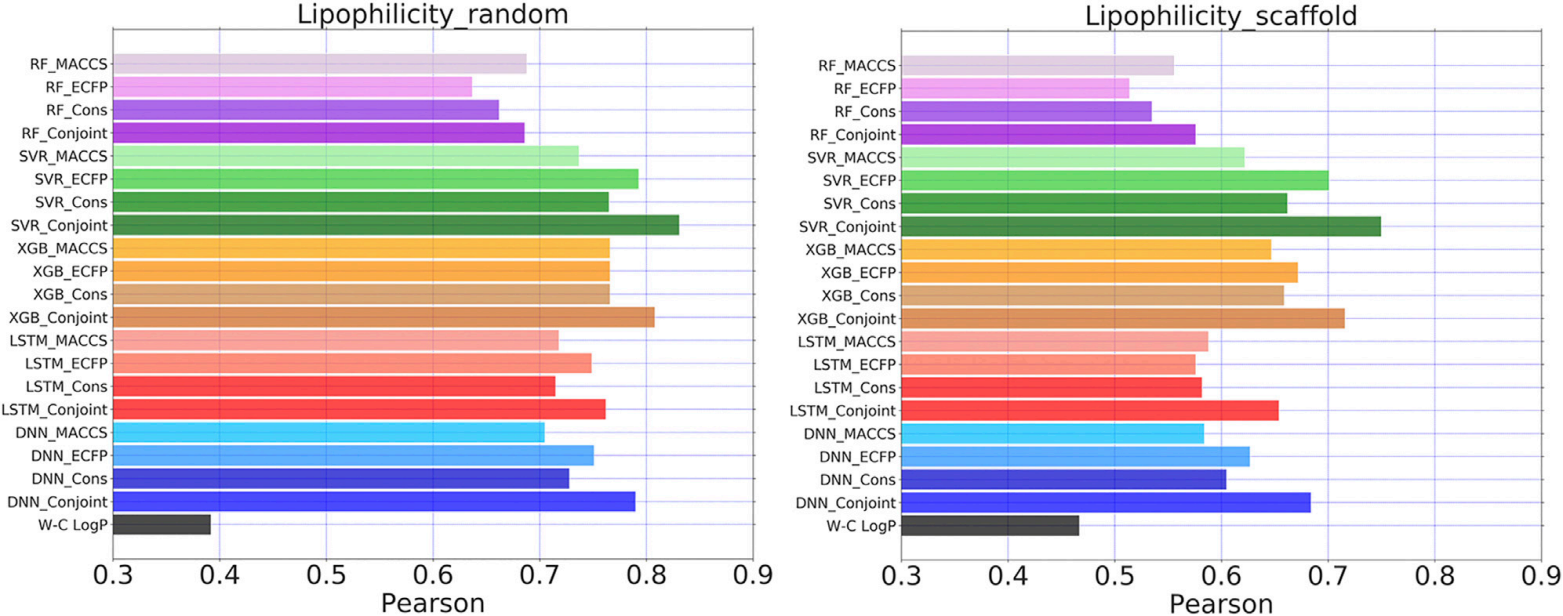
Pearson coefficients for Lipophilicity dataset calculated by five learning algorithms using MACCS keys, ECFP, consensus model and the conjoint fingerprints. The similar set of color represents one learning algorithm using different molecular fingerprints. Wildman-Crippen logP labeled as W-C logP.

Conjoint fingerprint is applicable to the Lipophilicity dataset from MoleculeNet. For all of five examined ML/DL methods, the predicted performance was improved by using conjoint fingerprints as shown in Figure 8. The predicted Pearson coefficient exceeded 0.8 by using SVR and XGBoost. The results were compared with one available computational method, SlogP computed by Wildman-Crippen logP prediction approach. On the same test set, the ML/DL methods outperformed over Wildman-Crippen logP computation method when using the current dataset. Wildman-Crippen logP reproduced different Pearson coefficient on the different split subset, implying that Wildman-Crippen logP computational method may also depend on the training dataset.

We also noticed that random splitting led to better performance of ML/DL methods than scaffold splitting. This is consistent with previous conclusions that substructure-based fingerprints likely result in better performance during random splitting than scaffold splitting. Scaffold splitting attempts to separate different chemical scaffold molecules into different subsets. Therefore, scaffold splitting can reveal the true learning abilities of ML/DL methods. In Figure 8, the Pearson coefficient difference between conjoint fingerprints and consensus model become more obvious, suggesting that the superiority of conjoint fingerprint over the consensus model. The result reminds us that we can quickly evaluate prediction quality by checking the substructure similarity between the training dataset and the test samples during practical applications.

**FIGURE 9:**
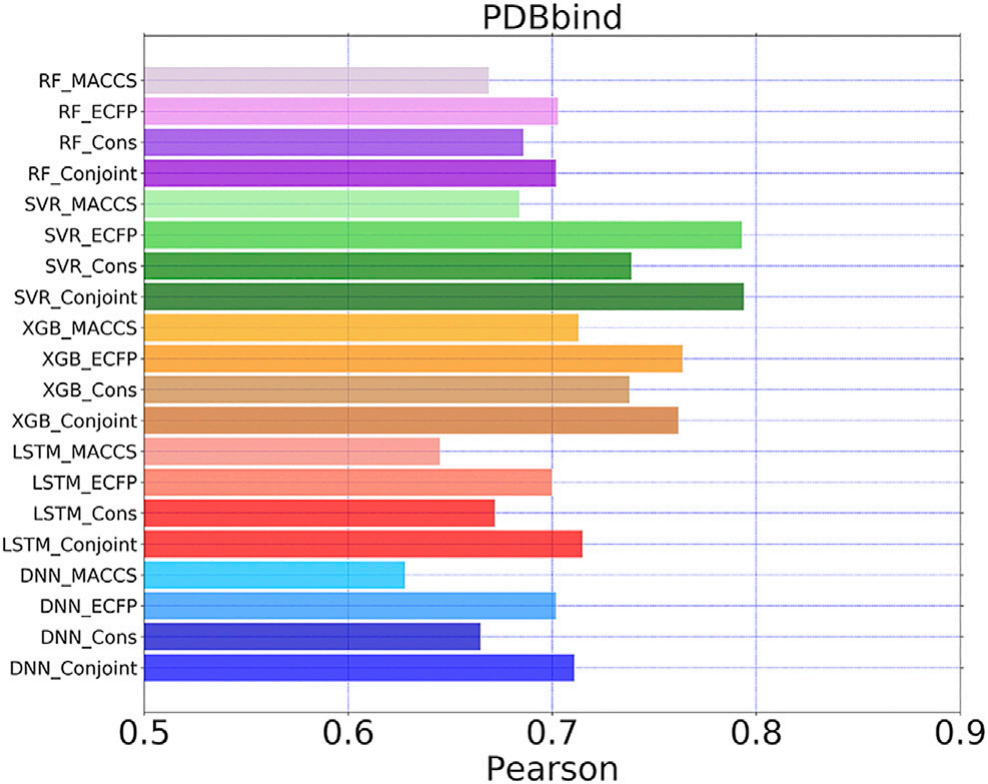
Pearson coefficients for PDBbind dataset calculated by five learning algorithms using MACCS keys, ECFP, consensus model and the conjoint fingerprints. The similar set of color represents one learning algorithm using different molecular fingerprints.

To demonstrate the generalizability of the proposed conjoint fingerprint, we conducted the regression task for PDBbind dataset. The Pearson coefficient between predicted and experimental pKi was computed for each ML/DL methods using MACCS keys, ECFP and conjoint fingerprints. Among evaluated methods, RF, SVR, and XGBboost produced the similar Pearson coefficient for ECFP and conjoint fingerprint as shown in Figure 9. LSTM and DNN lead to a higher Pearson coefficient for conjoint fingerprint than MACCS keys or ECFP. The Pearson coefficients obtained from conjoint fingerprints were higher than that obtained from the consensus model, implying that the combination of fingerprints can at least act as an alternative approach to the consensus model. The best predicting performance was achieved by the pairing of SVR and conjoint fingerprint, reaching the highest Pearson coefficient of 0.74, which is comparable to the predicted result with the grid featurization (Xie et al., 2020). Therefore, the conjoint fingerprint also contributed to the improved predicting performance in the regression task for PDBbind. The combination of two fingerprints will embody the information from each fingerprint. Without feature engineering, that taking all the combined fingerprints as the input for the ML/DL methods will provide more information while it also brings challenges for ML/DL at the meantime. Therefore we should select the matched ML/DL methods for the conjoint fingerprint via trial and error process. We believed that more improvement can be realized after optimizing hyperparameters for each ML/DL methods.

From our evaluation, we can notice that combining two types of fingerprints can obtain improved predicting performance than consensus model. Our manuscript acted as the preliminary demonstration on how to select multi-dimensional molecular fingerprints with matched ML/DL methods to circumvent feature selection. The combining scheme can be generally extended to other types of molecular descriptors and fingerprints. A rigorous evaluation of the conjoint fingerprints to check whether the conjoint fingerprint's superiority is statistically significant will be conducted in the future work.

## Discussion

When developing molecular representations, molecular descriptors have been optimized for the specific applications. Recombination of different types of molecular descriptors would be a convenient forward way to improved performance, especially for general users with no knowledge of molecular descriptor design. From our evaluation, we can see that conjoint fingerprint can improve predictive accuracy and reduce training cycles by leveraging automatic feature learning ability of deep learning. Using conjoint fingerprints, SVR and XGBoost achieved the comparative performance as that of LSTM and DNN. In applications, the choice of machine learning or deep learning depended on the task. The standalone featurization may inherently cover parts of information in the chemical molecules and thus the combination of accessible fingerprints would improve the predictive power of deep learning. The following points of view can be considered to improve predictive performance of deep learning when using conjoint fingerprints.

(1) Both MACCS keys and ECFP have been well documented in open-source software and thus other researchers can adopt them in their researches, which should facilitate applications of deep learning. Besides, we have witnessed great development in novel types of molecular descriptors in the last decade. Besides MACCS keys and ECFP, three dimensional types of ECFP (Axen et al., 2017), molecular graph convolutions (Kearnes et al., 2016) and atomic convolutional networks (Gomes et al., 2017) have been developed. The conjoint fingerprints can be built from other types of molecular descriptors besides the substructure based fingerprints. For example, conjoint fingerprints can be extended to include atomic or fragment-based molecular descriptors in the future work. Each new types of molecular descriptors show different merits. If they can provide open-source tools, it is worthy of conducting systematic search to find out the optimal combination of different types of molecular descriptors.
(2) For architectures of neural networks, convolutional neural networks and recurrent neural networks present as another exciting starting point to improve predictive performance of deep learning. Deep learning uses the hierarchical learning of representations (Zeiler and Fergus, 2014). The lowest layers of neural networks learn simple features that will be used to build higher order information along with their propagation through the networks. The informative features can be captured during hidden layers by automatically constructing one intermediate feature space. Deep learning will be expected reduce tedious works on intricate feature engineering. Experts from computer or related fields can provide more valuable insights if they have access to structural, topological and graphical fingerprints and other powerful deep learning architectures by following current protocol.

## Conclusion

We validated the impact of the conjoint fingerprints on three well established machine learning methods and two emerging deep learning methods, including RF, SVR, XGBoost, LSTM, and DNN. Combining MACCS keys with ECFP achieved complementarity in substructural and topological fingerprints, which can be processed by machine learning and deep learning algorithms to find the inherent rules between the demanded activity/property and their structures of drug molecules. Our results demonstrated that the conjoint fingerprints achieved the least loss and the highest Pearson coefficients than that of each standalone fingerprint for SVR, XGBoost, LSTM, and DNN, even surpassing the consensus model. By complementarily combining two types of fingerprints, boosted performance can be achieved than that of using single molecular descriptor. The proposed conjoint fingerprint scheme can be generally extended to other types of molecular descriptor. We anticipate that our proposed conjoint scheme would invoke following studies by integrating structural, topological or spatial fingerprints in deep learning area.

## Data Availability Statement

The datasets presented in this study can be found in online repositories. The names of the repository/repositories and accession numbers can be found below: https://github.com/xlxgit/AlogP-DL.git, github.

## Author Contributions

LiX and LeX contributed equally. The manuscript was written through contributions of all authors. All authors have given approval to the final version of the manuscript.

## Funding

This work was supported by the fund of the Natural Science Foundation of Jiangsu Province (BK20191032 and BE2019650), the National Natural Science Foundation of China (22003020, 12074151, and 81803430), Changzhou Sci. and Tech. Program (CJ20200045), and open funding from Jiangsu Sino-Israel Industrial Technology Research Institute (JSIITRI202009).

## Conflict of Interest

The authors declare that the research was conducted in the absence of any commercial or financial relationships that could be construed as a potential conflict of interest.
