# Supplementary material for: Improvement of Prediction Performance With Conjoint Molecular Fingerprint in Deep Learning
Source: Front Pharmacol. 2020 Dec 18;11:606668. doi: 10.3389/fphar.2020.606668 (PMC7819282; doi:10.3389/fphar.2020.606668)
Supplement: Supplementary file 1 [file datasheet1.docx]

Supplementary Material


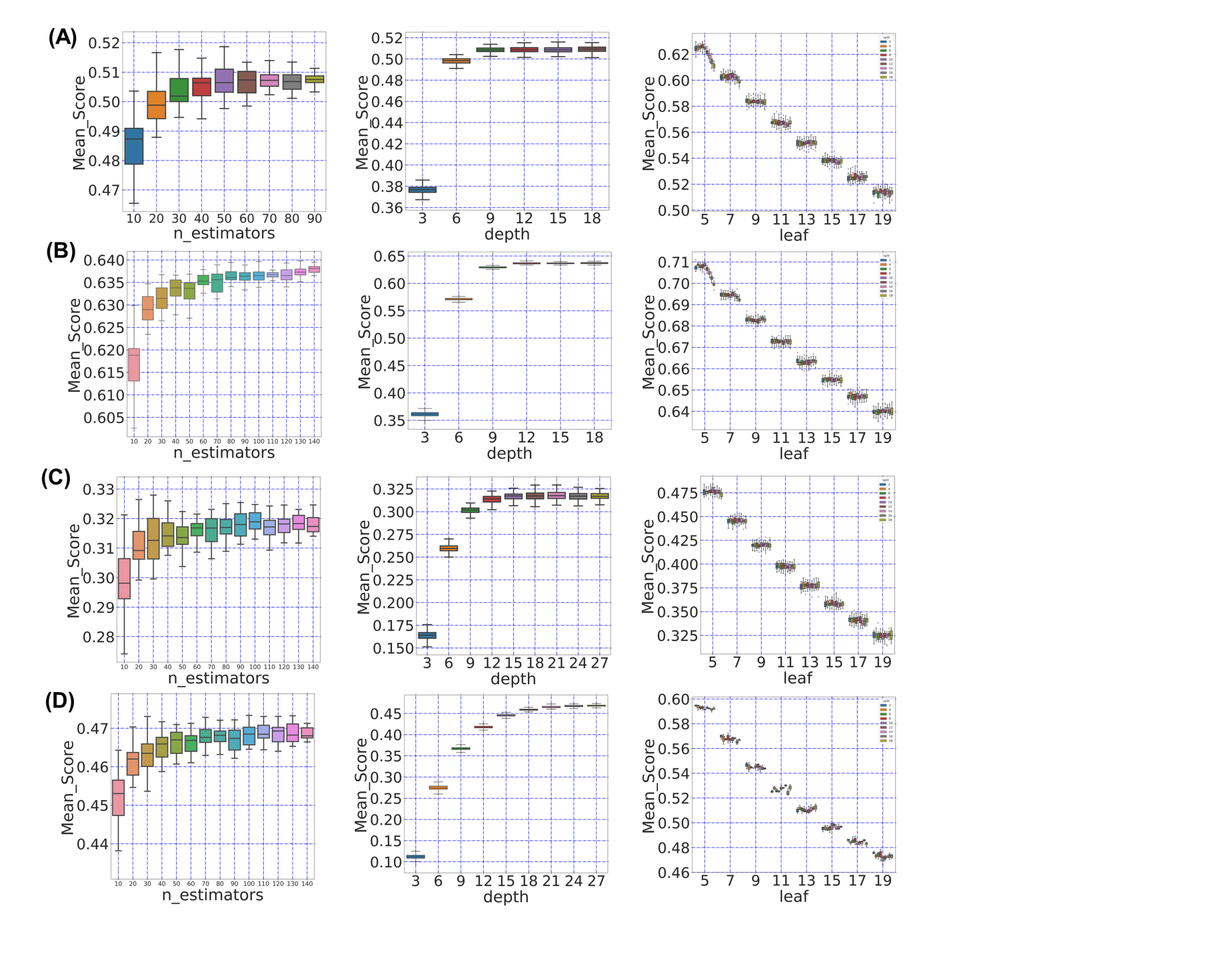


**Supplementary Figure 1**. The mean score of RF when using MACCS keys and ECFP during tuning hyperparameters. (A) Mean score of RF using MACCS keys for “Approved” subset; (B) Mean score of RF using MACCS keys for “All” subset; (C) Mean score of RF using ECFP for “Approved” subset; (D) Mean score of RF using ECFP for “All” subset.


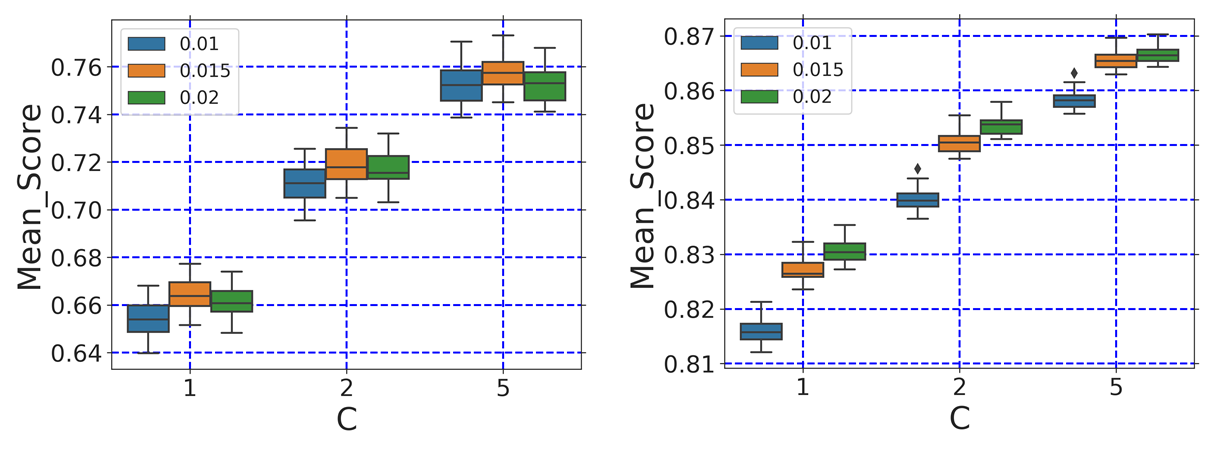


**Supplementary Figure 2.** The mean scores of SVR trained with ECFP during tuning hyperparameters. (A) Mean score of SVR for “Approved” subset; (B) Mean score of SVR for “All” subset.

**
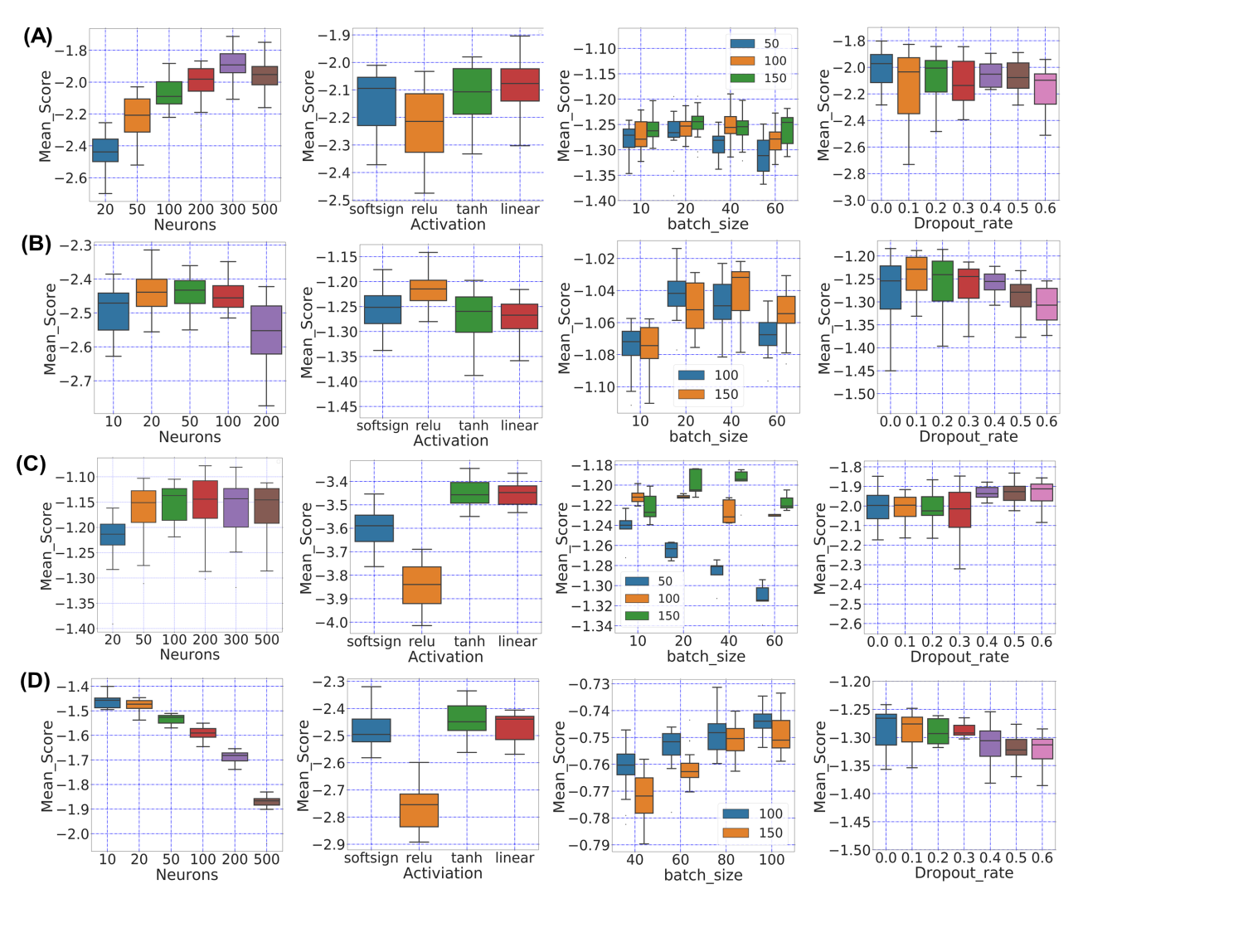
**

**Supplementary Figure 3**. The mean scores of DNN trained with MACCS keys and ECFP during tuning hyperparameters. (A) Mean score of DNN using MACCS keys for “Approved” subset; (B) Mean score of DNN using MACCS keys for “All” subset; (C) Mean score of DNN using ECFP for “Approved” subset; (D) Mean score of DNN using ECFP for “All” subset.


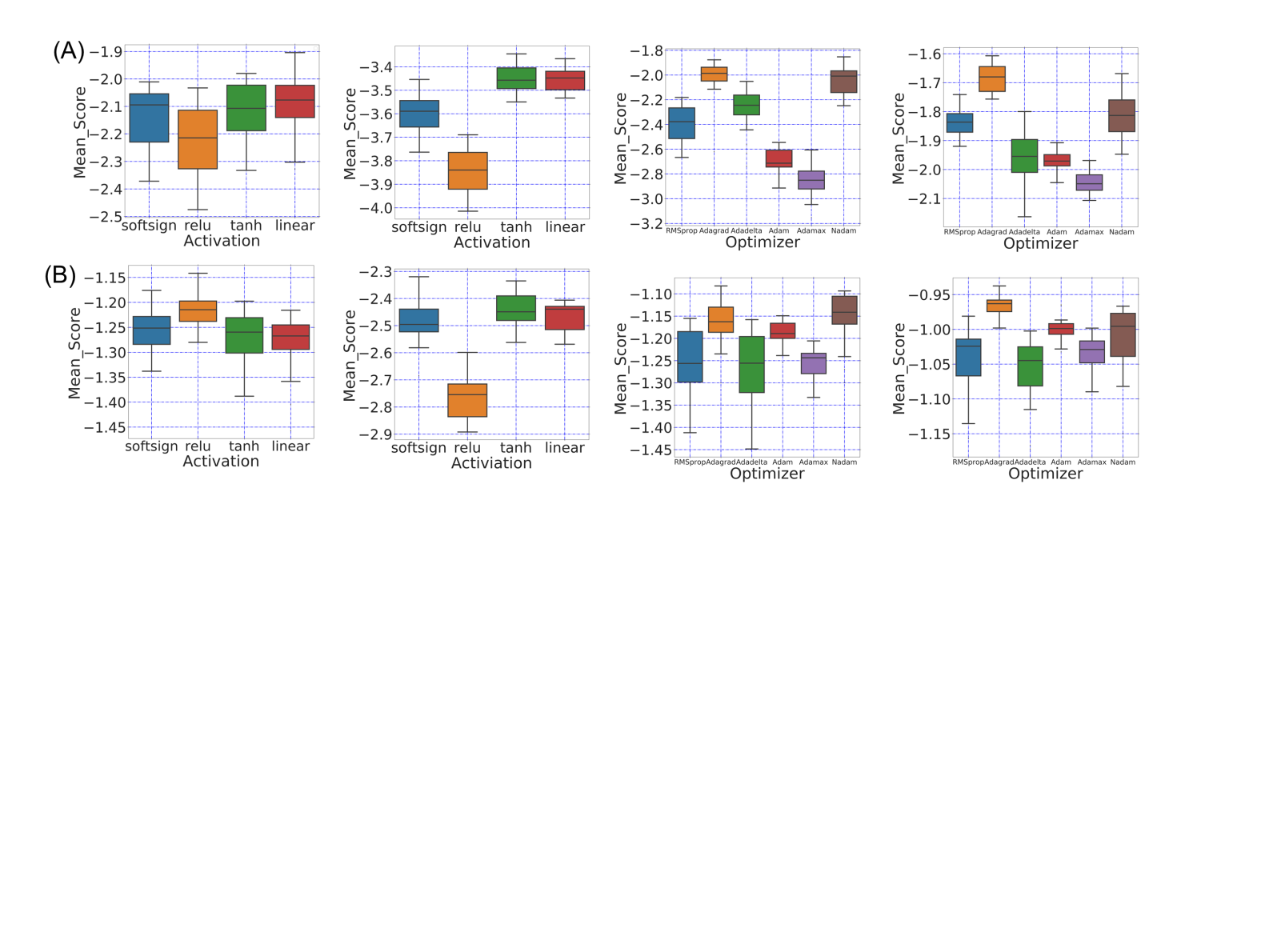


**Supplementary Figure 4**. The mean scores of LSTM trained with MACCS keys and ECFP during tuning hyperparameters. (A) Mean score of LSTM for “Approved” subset; (B) Mean score of LSTM for “All” subset.

**Table S1.** The selected hyperparameters tuned based on grid search method for DNN.

|  | Approved | | All | |
| --- | --- | --- | --- | --- |
|  | **MACCS** | **ECFP** | **MACCS** | **ECFP** |
| neurons | 300 | 50 | 100 | 10 |
| Batch size | 20 | 40 | 20 | 100 |
| epochs | 150 | 150 | 150 | 100 |
| dropout | 0.2 | 0.6 | 0.1 | 0.1 |
| activation | linear | linear | relu | linear |

**Table S2.** The selected hyperparameters tuned based on grid search method for LSTM.

|  | Approved | | All | |
| --- | --- | --- | --- | --- |
|  | **MACCS** | **ECFP** | **MACCS** | **ECFP** |
| Optimizer | Adagrad | Adagrad | Nadam | Adagrad |
| activation | linear | linear | Linear | linear |

**Table S3**. The summarized Pearson coefficients for training and test subset of all evaluated methods.

|  |  | **Approved** | | **All** | |
| --- | --- | --- | --- | --- | --- |
| **Methods** | **fingerprint** | **train** | **test** | **train** | **test** |
| **RF** | **MACCS** | 0.905 | 0.817 | 0.922 | 0.892 |
| **RF** | **ECFP** | 0.855 | 0.787 | 0.887 | 0.866 |
| **RF- Cons** | **MACCS+ECFP** | 0.902 | 0.827 | 0.920 | 0.899 |
| **RF** | **conjoint** | 0.831 | 0.764 | 0.873 | 0.856 |
| **RF** | **Conjoint-opt** | 0.844 | 0.767 | 0.874 | 0.857 |
| **SVR** | **MACCS** | 0.949 | 0.865 | 0.955 | 0.926 |
| **SVR** | **ECFP** | 0.985 | 0.869 | 0.990 | 0.942 |
| **SVR-Cons** | **MACCS+ECFP** | 0.977 | 0.891 | 0.982 | 0.953 |
| **SVR** | **conjoint** | 0.996 | 0.905 | 0.984 | 0.959 |
| **SVR** | **Conjoint-opt** | 0.990 | 0.906 | 0.995 | 0.962 |
| **XGB** | **MACCS** | 0.992 | 0.879 | 0.980 | 0.932 |
| **XGB** | **ECFP** | 0.975 | 0.881 | 0.966 | 0.930 |
| **XGB-Cons** | **MACCS+ECFP** | 0.989 | 0.904 | 0.980 | 0.949 |
| **XGB** | **conjoint** | 0.989 | 0.908 | 0.982 | 0.952 |
| **XGB** | **Conjoint-opt** | 0.995 | 0.908 | 0.990 | 0.950 |
| **LSTM** | **MACCS** | 0.970 | 0.863 | 0.961 | 0.916 |
| **LSTM** | **ECFP** | 0.975 | 0.858 | 0.975 | 0.914 |
| **LSTM-Cons** | **MACCS+ECFP** | 0.981 | 0.900 | 0.982 | 0.942 |
| **LSTM** | **conjoint** | 0.984 | 0.903 | 0.988 | 0.949 |
| **LSTM** | **Conjoint-opt** | 0.982 | 0.904 | 0.988 | 0.949 |
| **DNN** | **MACCS** | 0.973 | 0.865 | 0.975 | 0.912 |
| **DNN** | **ECFP** | 0.976 | 0.882 | 0.980 | 0.935 |
| **DNN-Cons** | **MACCS+ECFP** | 0.982 | 0.905 | 0.985 | 0.948 |
| **DNN** | **conjoint** | 0.980 | 0.910 | 0.976 | 0.951 |
| **DNN** | **Conjoint-opt** | 0.977 | 0.908 | 0.986 | 0.957 |

**Table S4**. The loss of LSTM and DNN during training and validation.

|  |  | Approved |  | All |  |
| --- | --- | --- | --- | --- | --- |
| Methods | **fingerprint** | **train** | **validation** | **train** | **Validation** |
| LSTM | **MACCS** | 0.085 | 1.338 | 0.283 | 0.866 |
| LSTM | **ECFP** | 0.259 | 1.205 | 0.181 | 0.901 |
| LSTM-Cons | **MACCS+ECFP** | 0.366 | 3.002 | 0.381 | 1.790 |
| LSTM | **conjoint** | 0.011 | 0.827 | 0.018 | 0.583 |
| DNN | **MACCS** | 0.142 | 1.190 | 0.194 | 1.019 |
| DNN | **ECFP** | 0.347 | 1.217 | 0.367 | 0.798 |
| DNN-Cons | **MACCS+ECFP** | 1.340 | 2.927 | 0.467 | 1.772 |
| DNN | **conjoint** | 0.170 | 0.789 | 0.284 | 0.656 |
